# Phosphatidylserine externalization and procoagulant activation of erythrocytes induced by *Pseudomonas aeruginosa* virulence factor pyocyanin

**DOI:** 10.1111/jcmm.12778

**Published:** 2016-01-19

**Authors:** Syed M. Qadri, David A. Donkor, Varsha Bhakta, Louise J. Eltringham‐Smith, Dhruva J. Dwivedi, Jane C. Moore, Laura Pepler, Nikola Ivetic, Ishac Nazi, Alison E. Fox‐Robichaud, Patricia C. Liaw, William P. Sheffield

**Affiliations:** ^1^ Centre for Innovation Canadian Blood Services Hamilton ON Canada; ^2^ Department of Pathology and Molecular Medicine McMaster University Hamilton ON Canada; ^3^ Thrombosis and Atherosclerosis Research Institute (TaARI) McMaster University Hamilton ON Canada; ^4^ Department of Medicine McMaster University Hamilton ON Canada; ^5^ Department of Biochemistry and Biomedical Sciences McMaster University Hamilton ON Canada

**Keywords:** *Pseudomonas aeruginosa*, pyocyanin, erythrocyte, phosphatidylserine, coagulation

## Abstract

The opportunistic pathogen *Pseudomonas aeruginosa* causes a wide range of infections in multiple hosts by releasing an arsenal of virulence factors such as pyocyanin. Despite numerous reports on the pleiotropic cellular targets of pyocyanin toxicity *in vivo*, its impact on erythrocytes remains elusive. Erythrocytes undergo an apoptosis‐like cell death called eryptosis which is characterized by cell shrinkage and phosphatidylserine (PS) externalization; this process confers a procoagulant phenotype on erythrocytes as well as fosters their phagocytosis and subsequent clearance from the circulation. Herein, we demonstrate that *P. aeruginosa* pyocyanin‐elicited PS exposure and cell shrinkage in erythrocyte while preserving the membrane integrity. Mechanistically, exposure of erythrocytes to pyocyanin showed increased cytosolic Ca^2+^ activity as well as Ca^2+^‐dependent proteolytic processing of μ‐calpain. Pyocyanin further up‐regulated erythrocyte ceramide abundance and triggered the production of reactive oxygen species. Pyocyanin‐induced increased PS externalization in erythrocytes translated into enhanced prothrombin activation and fibrin generation in plasma. As judged by carboxyfluorescein succinimidyl‐ester labelling, pyocyanin‐treated erythrocytes were cleared faster from the murine circulation as compared to untreated erythrocytes. Furthermore, erythrocytes incubated in plasma from patients with *P. aeruginosa* sepsis showed increased PS exposure as compared to erythrocytes incubated in plasma from healthy donors. In conclusion, the present study discloses the eryptosis‐inducing effect of the virulence factor pyocyanin, thereby shedding light on a potentially important mechanism in the systemic complications of *P. aeruginosa* infection.

## Introduction

The opportunistic pathogen *Pseudomonas aeruginosa* causes a wide range of infections in humans and is responsible for the progressive loss of pulmonary function in patients with cystic fibrosis 1, 2. *P. aeruginosa* is also a primary cause of sepsis and mortality in immunocompromised individuals 3, 4. It can infect hosts of multiple phylogenetic backgrounds and has a complex pathophysiology of infection because of the release of a large arsenal of virulence factors 5. Toxic metabolites produced by *P. aeruginosa* include alkaline proteases, elastase, rhamnolipids and phenazines 2, 6, 7. Phenazines comprise a large family of quorum‐sensing tricyclic molecules such as pyocyanin which have a high diffusion capacity 8. Pyocyanin (*N*‐methyl‐1‐hydroxyphenazine), a redox‐active secondary metabolite, is toxic for both eukaryotic and prokaryotic cells and is a major virulence factor in *P. aeruginosa* infection in humans 9, 10, 11.

Pyocyanin influences a wide array of cellular functions by targeting pathways involved in the cell cycle, Ca^2+^ homeostasis, mitochondrial electron transport and respiration, protein sorting as well as vesicle transport 5, 11, 12. It further compromises cellular energy balance 5, antagonizes nitric oxide activity 13 and is a powerful modulator of eicosanoid biosynthesis 14, 15. Cytotoxic ramifications of pyocyanin include autophagy and apoptosis of various cell types 10, 16, 17, 18, 19, 20, 21. Putative mechanisms involved in pyocyanin‐elicited cytotoxicity encompass caspase activation, mitochondrial membrane permeabilization, redox‐sensitive lysosomal destabilization, DNA damage and signal transduction involving the phosphoinositide 3‐kinase pathway and its downstream effector Akt as well as the mitogen‐activated protein kinase ERK1/2 17, 20, 21, 22. Moreover, pyocyanin was shown to foster the activation of transcription factors such as Nrf2 and NF‐κB 22, 23.

Biological sequelae of pyocyanin‐induced toxicity *in vivo* are not completely understood. Remarkably, increased pyocyanin production has been implicated as a pivotal mechanism involved in causing increased lethality in mice due to *P. aeruginosa* sepsis 24 potentially suggesting similar detrimental systemic effects of this virulence factor in *P. aeruginosa* bacteraemia in humans. Although blood concentrations of pyocyanin during *P. aeruginosa* sepsis have not been reported, the concentrations of pyocyanin have been shown to approach >100 μM in the sputum of cystic fibrosis patients 25. Despite numerous reports on the pleiotropic cellular targets of pyocyanin toxicity, its impact on erythrocytes remains elusive.

In analogy to apoptosis of nucleated cells, erythrocytes may undergo programmed cell death or eryptosis, which is characterized by cell shrinkage and phospholipid scrambling of the cell membrane 26, 27. The eryptosis machinery includes activation of redox‐sensitive Ca^2+^‐permeable cation channels resulting in Ca^2+^ entry, activation of Ca^2+^‐sensitive K^+^ channels, exit of KCl with osmotically obliged water and, thus, cell shrinkage 27, 28. Cytosolic Ca^2+^ further activates erythrocyte scramblase and calpain resulting in phosphatidylserine (PS) externalization and membrane blebbing respectively 27. Eryptosis may further be orchestrated independently of cytosolic Ca^2+^ activity *via* caspases or sphingomyelinase activation that subsequently triggers ceramide formation 27. Phosphatidylserine‐exposing erythrocytes are rapidly phagocytosed and, thus, cleared from circulating blood 29. In addition, PS exposure confers a procoagulant phenotype on erythrocytes 30. Excessive eryptosis, thus, contributes to the pathogenesis of anaemia and thrombosis in systemic conditions associated with this phenomenon 27, 30.

Bacterial infections may lead to anaemia 31 and dysregulated coagulation 32 which, at least in part, may result from enhanced eryptosis 33. In the present study, we aimed to investigate whether *P. aeruginosa* pyocyanin impacts erythrocyte survival and, if so, to elucidate the underlying mechanisms.

## Materials and methods

### Erythrocytes, chemicals and patients

The use of leukoreduced erythrocytes obtained from healthy volunteer donors with informed consent was approved by the Canadian Blood Services Research Ethics Board (#2015.022). Phlebotomy and component production was done by the Canadian Blood Services Network Centre for Applied Development (netCAD, Vancouver, BC, Canada). Erythrocyte units were shipped to this laboratory using shipping containers validated to maintain internal temperature between 1 and 10°C and were refrigerated on receipt. Unless otherwise indicated, erythrocytes (haematocrit 0.4%) were incubated in Ringer's solution containing 125 mM NaCl, 5 mM KCl, 5 mM glucose, 32 mM HEPES, 1 mM Mg_2_SO_4_, 1 mM CaCl_2_ (pH 7.4). Where indicated, 0–100 μM pyocyanin (Sigma‐Aldrich, St. Louis, MO, USA), 0–100 μM 1‐hydroxyphenazine (TCI America, Portland, OR, USA), 0–100 μM phenazine‐1‐carboxylic acid (Apollo Scientific, Stockport, United Kingdom) or the pancaspase inhibitor Z‐VAD‐FMK (10 μM; R&D Systems, Minneapolis, MN, USA) was added or extracellular Ca^2+^ removed and replaced with 1 mM ethylene glycol tetraacetic acid (EGTA). Erythrocytes were also incubated in plasma obtained from patients diagnosed with *P. aeruginosa* sepsis, who were enrolled in the DYNAMICS (DNA as a Prognostic Marker in Intensive Care Unit Patients Study) registered clinical trial NCT01355042. This study was approved by the Research Ethics Board of McMaster University and the Hamilton Health Sciences, Hamilton, Ontario, Canada (REB approval November 2010). Written informed consent was obtained from the patient (or substitute decision‐maker). The DYNAMICS database was searched for patients with severe sepsis and *P. aeruginosa* positive cultures and frozen plasma samples were retrieved from the biobank for study. Clinical features of the recruited *P. aeruginosa* sepsis patients are shown in Table 1. In control experiments, erythrocytes were also incubated in plasma obtained from residual blood samples from healthy volunteer donors in a study approved by the Research Ethics Board of McMaster University and the Hamilton Health Sciences, Hamilton, Ontario, Canada; these samples were provided blinded with no access to any information that could have identified the donors.

**Table 1:** Clinical characteristics of patients with *Pseudomonas aeruginosa* sepsis

No.	Age	Sex	Septic focus	Apache II score	ICU stay (days)	Outcome
1	75	M	Pneumonia	36	65	Alive
2	38	F	Blood	22	13	Expired
3	58	M	Pneumonia	25	23	Expired
4	81	M	Biliary sepsis	40	13	Alive
5	88	F	Pneumonia	13	7	Alive
6	64	M	Pneumonia	14	8	Alive

### Flow cytometry

After treatment of erythrocytes under the respective conditions, erythrocytes were washed twice in Ringer's solution prior to Fluorescence Activated Cell Sorting (FACS) analysis. Fluorescence intensity was measured in the FL1 channel with an excitation wavelength of 488 nm and an emission wavelength of 530 nm using EPICS XL‐MCL (Beckman Coulter, Mississauga, ON, Canada) flow cytometer. Erythrocyte cell volume was estimated using forward scatter analysis independently of fluorescence parameters. To determine PS exposure, erythrocytes were stained with Annexin‐V‐FLUOS (1:1000; Roche Diagnostics, Laval, QC, Canada) in Ringer's solution containing an additional 4 mM CaCl_2_ for 15 min. at 37°C. To estimate cytosolic Ca^2+^ activity, erythrocytes were stained with Fluo3/AM (2 μM in Ringer's solution; Biotium Hayward, CA, USA) for 15 min. at 37°C. To determine ceramide abundance, erythrocytes were stained for 1 hr at 37°C with 1 μg/ml anti‐ceramide antibody (clone MID 15B4; 1:5; Enzo, Farmingdale, NY, USA) diluted in PBS containing 0.1% bovine serum albumin (BSA). After washing twice with PBS‐BSA and staining with FITC‐conjugated goat antimouse IgG/IgM specific antibody (1:50; BD Biosciences, Mississauga, ON, Canada) for 30 min. at 37°C, the erythrocytes were subsequently washed three times with PBS‐BSA prior to FACS analysis. To estimate Reactive Oxygen Species (ROS) production, erythrocytes suspended in Ringer's solution were stained with the non‐polar and non‐fluorescent probe 2′,7′,‐dichlorodihydrofluorescein diacetate (10 μM; Sigma‐Aldrich) for 30 min. at 37°C. Erythrocytes were then washed twice in Ringer's solution and finally resuspended again in Ringer's solution. The geomean of DCF‐dependent fluorescence was quantified in FL1 channel using FACS analysis. All data generated using FACS analysis were analysed using FlowJo^®^ software (FlowJo LLC, Ashland, OR, USA).

### Estimation of haemolysis

After treatment of erythrocytes (0.4%) with different concentrations of pyocyanin in Ringer's solution for 48 hrs, the erythrocytes were centrifuged (3 min. at 400 × g) and cell‐free haemoglobin concentration was determined in the supernatant using a human haemoglobin ELISA kit (Abcam, Toronto, ON, Canada) according to the manufacturer's instructions.

### Immunoblotting

Erythrocytes (5% haematocrit) were incubated with different concentrations of pyocyanin for 48 hrs or 10 μM ionomycin for 30 min. The erythrocytes were then washed twice in PBS and subsequently subjected to lysis using a lysis buffer containing 50 mM Tris‐HCl, pH 7.5, 150 mM NaCl, 1% Triton X‐100, 0.5% SDS, 0.4% β‐mercaptoethanol, and protease inhibitor cocktail (Roche). The samples were then mixed with loading buffer (Roti‐Load 1; Roth, Carl Roth GmbH, Karlsruhe, Germany), boiled at 95°C for 5 min. and resolved by 10% SDS‐PAGE. For immunoblotting, proteins were electrotransferred onto polyvinylidene fluoride (PVDF) membrane and blocked with 5% non‐fat milk in TBS‐0.1% Tween 20 (TBST) at room temperature for 1 hr. The membrane was incubated at 4°C overnight with rabbit polyclonal anti‐calpain 1 antibody which detects both latent and amino‐processed calpain 1 (1:1000; 82 kDa; Abcam). After washing with TBST the blots were incubated with secondary horseradish peroxidase‐ (HRP‐) conjugated anti‐rabbit antibody (1:5000; Jackson Immunoresearch, West Grove, PA, USA) for 1 hr at room temperature. After washing, antibody binding was chromogenically detected using diaminobenzamidine (Sigma‐Aldrich).

### Prothrombin activation assay

Prothrombin activation by eryptotic erythrocytes was determined using a previously described assay 30. Briefly, untreated (Control) and pyocyanin‐treated erythrocytes (4.5% haematocrit) incubated for 48 hrs were treated with human factor Xa (2 nM; Haematologic Technologies, Essex Junction, VT, USA), human factor Va (0.2 nM; Haematologic Technologies) and 2 mM CaCl_2_ for 3 min. at 37°C. The erythrocytes were then treated with prothrombin (1.4 μM; Haematologic Technologies) for 5 min. and the reaction was stopped by the addition of 10 mM ethylenediaminetetraacetic acid. The samples were then centrifuged (3 min. at 400 × g), diluted fivefold and kinetically evaluated at 405 nm following addition of the chromogenic substrate S2238 (100 μM; Diapharma, West Chester, OH, USA). As a negative control, the coagulation factors and other agents were added to the solution in the absence of erythrocytes.

### Recalcification clotting test

To evaluate the procoagulant properties of pyocyanin‐treated erythrocytes in plasma, 50 μl of erythrocytes were added to 50 μl of human plasma 37°C in the absence of any other exogenous phospholipid source. Clotting time was determined after the addition of 10 mM CaCl_2_ in an electromagnetic coagulometer (ST art4 anaylzer; Diagnostica Stago, Asnieres, France).

### Determination of the *in vivo* clearance of fluorescence‐labelled erythrocytes

The *in vivo* clearance of fluorescence‐labelled erythrocytes in mice was determined as described previously 29. CD1 mice (Charles River, Montreal, QC, Canada) weighing approximately 30 g after acclimatization were used for erythrocyte clearance experiments. All procedures complied with Canadian Council on Animal Care guidelines and an animal utilization protocol (AUP#12‐07‐30) approved by the Animal Research Ethics Board of the Faculty of Health Sciences, McMaster University. Isolated murine erythrocytes obtained from 200 μl blood from donor mice as a terminal procedure were incubated for 12 hrs in the absence or presence of 50 μM pyocyanin. Cells were then fluorescence‐labelled by staining the cells with carboxyfluorescein‐diacetate‐succinimidyl‐ester (CFSE; 5 μM; Invitrogen, Carlsbad, CA, USA) in phosphate‐buffered saline (PBS) and incubated for 30 min. at 37°C. The erythrocytes were then washed twice in PBS supplemented with 1% Fetal Calf Serum (FCS) and resuspended in pre‐warmed Ringer solution and 100 μl of labelled erythrocytes were infused into the tail vein of recipient mice. Blood was retrieved from tail veins and CFSE‐dependent fluorescence was quantified using flow cytometry 5, 30 and 60 min. after infusion of labelled erythrocytes. The percentage of CFSE‐positive erythrocytes was calculated as the percentage of the total labelled fraction determined 5 min. after infusing the labelled erythrocytes.

### Confocal microscopy

Phosphatidylserine exposure was visualized by staining 50 μl of erythrocytes with Annexin‐V‐FLUOS (1:100). Erythrocytes were then washed twice in Ringer's solution containing 4 mM CaCl_2_ and resuspended in the same solution and visualized using a Zeiss LSM 510 META confocal laser scanning microscope (Carl Zeiss Inc., Thornwood, NY, USA) with a C‐Apochromat 63×/1.2 W corr objective. For the detection of PS exposure and CFSE‐dependent fluorescence of erythrocytes from murine spleens, the spleens were homogenized mechanically in cold PBS. After centrifugation, the cell pellet was resuspended in Ringer's solution containing 5 mM CaCl_2_ and stained with annexin V‐APC (1:20; BD Biosciences). The suspension was then transferred onto a glass slide and images were taken as above.

### Statistics

Data are expressed as arithmetic means ± S.E.M., and statistical analyses were performed using paired or unpaired Student's *t*‐test or anova as appropriate. *P* < 0.05 was considered statistically significant.

## Results

**Figure 1:**
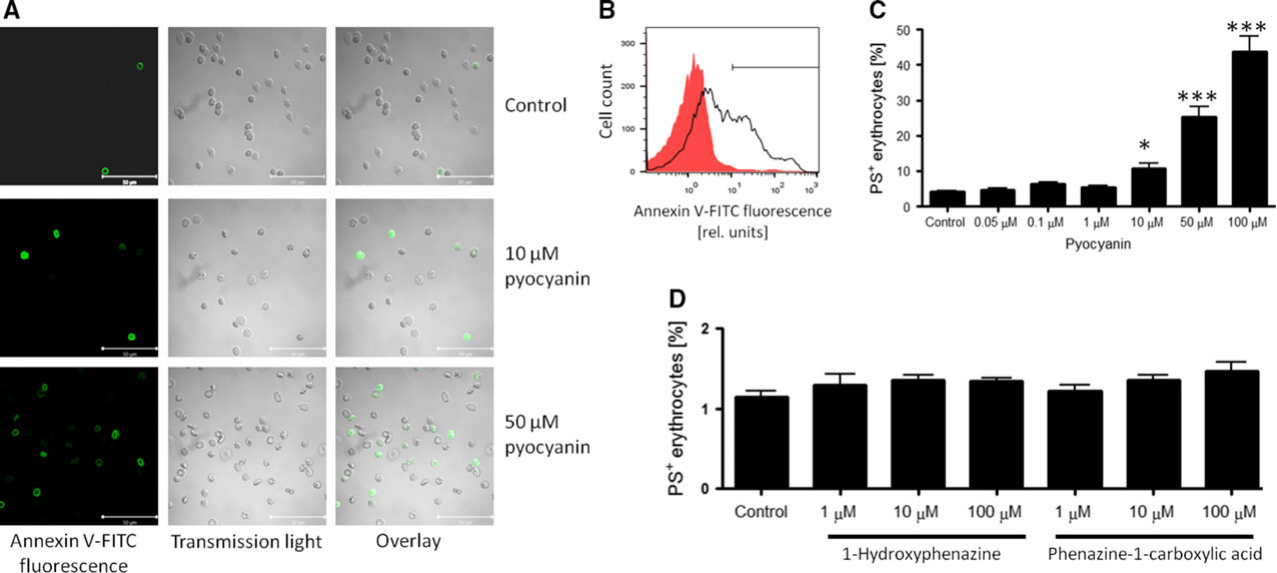
Effect of *Pseudomonas aeruginosa* pyocyanin on erythrocyte membrane phospholipid asymmetry. (**A**) Representative confocal microscopy images of FITC‐dependent annexin V fluorescence of erythrocytes incubated for 48 hrs in the absence (Control) and in the presence of pyocyanin (10 and 50 μM). For comparison, images were taken under transmission light and overlaid with fluorescence images. Original histogram (*red shadow*: Control, *black line*: 50 μM pyocyanin; **B**) and means ± S.E.M. (**C**) of percentage of PS exposing erythrocytes (*n* = 8–13) following 48‐hr incubation with 0–100 μM pyocyanin. *,*** (*P* < 0.05, *P* < 0.001) from Control. (**D**) Means ± S.E.M. of percentage of PS exposing erythrocytes (*n* = 3) following 48‐hr incubation with 1‐hydroxyphenazine or phenazine‐1‐carboxylic acid (0–100 μM).

Firstly, we explored whether *P. aeruginosa* pyocyanin influences membrane phospholipid asymmetry and cell volume of erythrocytes, the defining morphological features of eryptosis. Fluorescence microscopy images show an increased number of annexin V positive erythrocytes following 48‐hr treatment with both 10 and 50 μM pyocyanin, reflecting increased PS exposure (Fig. 1A). FACS analysis was subsequently performed to quantify annexin V‐binding following pyocyanin treatment. As shown in Figure 1B and C, pyocyanin increased the percentage of annexin V positive erythrocytes, an effect reaching statistical significance at 10 μM pyocyanin. We further tested whether other *P. aeruginosa* phenazine derivatives such as 1‐hydroxyphenazine and phenazine‐1‐carboxylic acid similarly induced erythrocyte PS exposure. As shown in Figure 1D, treatment with either 1‐hydroxyphenazine or phenazine‐1‐carboxylic acid (1–100 μM) for 48 hrs did not significantly enhance the percentage of annexin V positive erythrocytes. These findings suggest that pyocyanin, and not related aromatic compounds, elicits a specific pro‐eryptotic effect.

**Figure 2:**
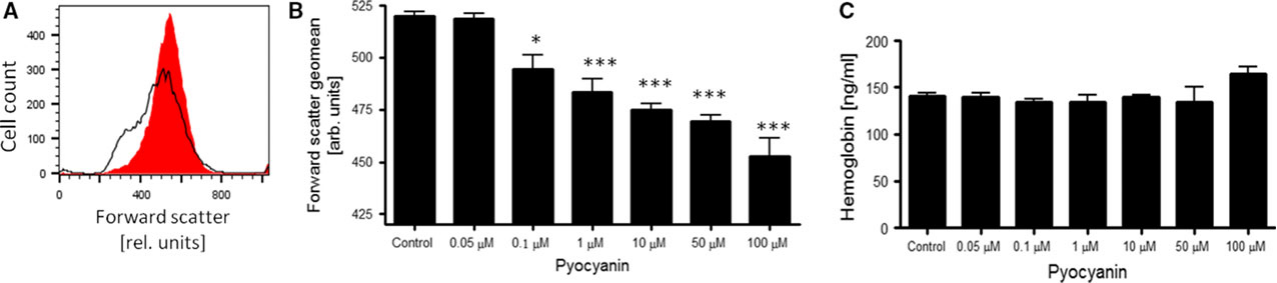
Effect of *Pseudomonas aeruginosa* pyocyanin on cell volume and membrane integrity of erythrocytes. Original histogram (*red shadow*: Control, *black line*: 50 μM pyocyanin; **A**) and means ± S.E.M. (**B**) of forward scatter geomean determined in erythrocytes (*n* = 8–13) following 48‐hr incubation with 0–100 μM pyocyanin. *,*** (*P* < 0.05, *P* < 0.001) from Control. (**C**) Means ± S.E.M. of haemoglobin concentration in supernatants of erythrocytes (*n* = 4) incubated for 48 hrs with 0–100 μM pyocyanin.

Forward scatter in FACS analysis was further employed to detect alterations in erythrocyte cell volume. As shown in Figure 2A and B, pyocyanin treatment decreased erythrocyte forward scatter, an effect reaching statistical significance at 0.1 μM pyocyanin. Pyocyanin, thus, triggered both PS externalization and cell shrinkage in erythrocytes. Further experiments explored whether pyocyanin treatment compromises the integrity of erythrocyte membrane. Quantification of haemoglobin released in the supernatant revealed that pyocyanin did not significantly alter erythrocyte membrane integrity until concentrations of 50 μM pyocyanin were reached. Pyocyanin tended to enhance haemolysis at a concentration of 100 μM, an effect, however, not reaching statistical significance (Fig. 2C).

**Figure 3:**
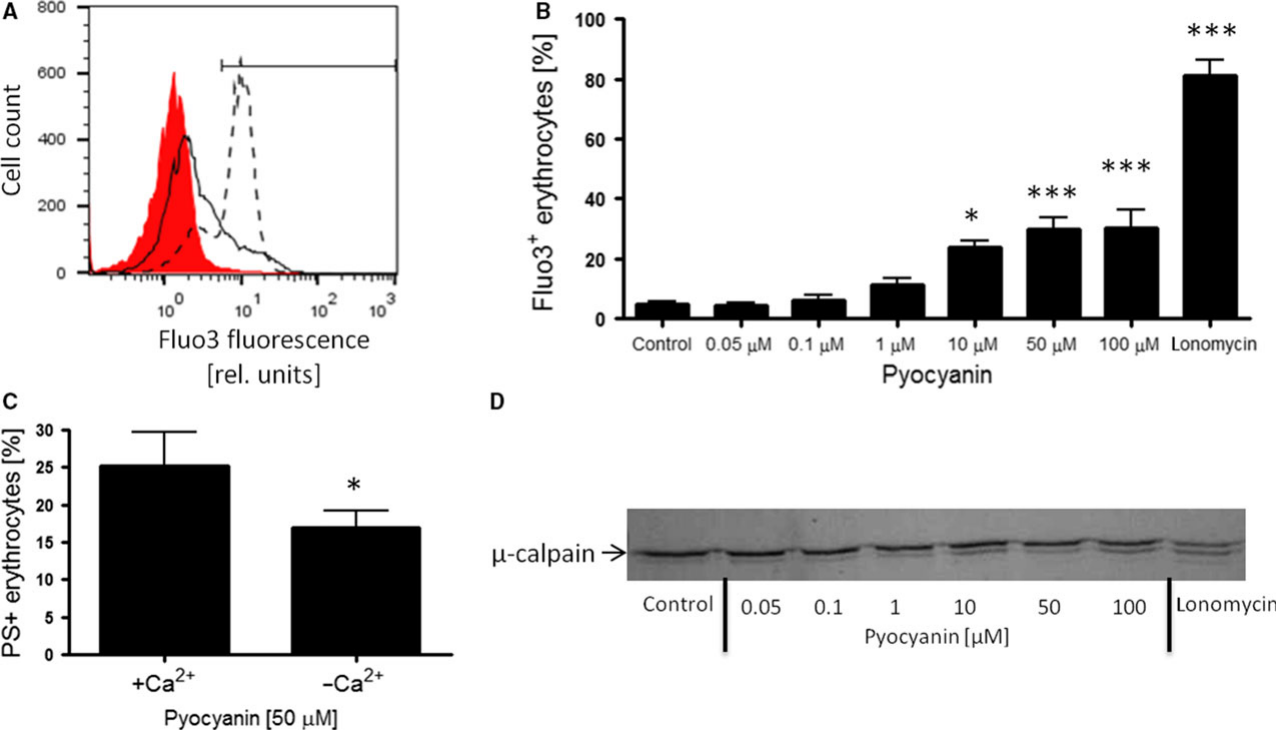
Effect of *Pseudomonas aeruginosa* pyocyanin on erythrocyte Ca^2+^ signalling. Original histogram (*red shadow*: Control, *black line*: 50 μM pyocyanin, *dashed line*: ionomycin; **A**) and means ± S.E.M. (**B**) of percentage of Fluo3 positive erythrocytes (*n* = 4–12) following 48‐hr incubation with 0–100 μM pyocyanin or ionomycin (10 μM for 30 min.). *,*** (*P* < 0.05, *P* < 0.001) from Control. (**C**) Means ± S.E.M. of percentage of PS exposing erythrocytes (*n* = 9) following 48‐hr incubation with 50 μM pyocyanin in the absence (−Ca^2+^) or in the presence (+Ca^2+^) of extracellular calcium. * (*P* < 0.05) from +Ca^2+^. (**D**) Proteolytic cleavage of μ‐calpain determined by immunoblotting (representative of three experiments) in erythrocytes following 48‐hr incubation with 0–100 μM pyocyanin or ionomycin (10 μM for 30 min.).

We then sought to elucidate the underlying mechanisms in pyocyanin‐induced breakdown of phospholipid asymmetry and cell shrinkage in erythrocytes. Cytosolic Ca^2+^ activity was determined using Fluo3 fluorescence in FACS analysis. As illustrated in Figure 3A and B, the percentage of Fluo3 positive erythrocytes was increased following treatment with pyocyanin, an effect reaching statistical significance at 10 μM. As a positive control, incubation of erythrocytes with the Ca^2+^ ionophore ionomycin increased the percentage of Fluo3 positive cells (Fig. 3A and B). Further experiments showed that in the absence of extracellular Ca^2+^, pyocyanin‐induced phospholipid scrambling was significantly blunted but not abolished, indicating that cytosolic Ca^2+^ activity indeed contributes to pyocyanin‐induced PS exposure (Fig. 3C). In addition to activation of Ca^2+^‐sensitive scramblase, cytosolic Ca^2+^ activity further elicits activation of the erythrocyte protease calpain 34. As depicted in Figure 3D, pyocyanin treatment elicited proteolytic cleavage of μ‐calpain which was more pronounced at higher pyocyanin concentrations. As a positive control, ionomycin treatment similarly enhanced proteolytic processing of μ‐calpain (Fig. 3D). These data indicate that Ca^2+^‐dependent signalling contributes to, but does not completely account for, pyocyanin‐induced eryptosis suggesting that other mechanisms may be operative.

**Figure 4:**
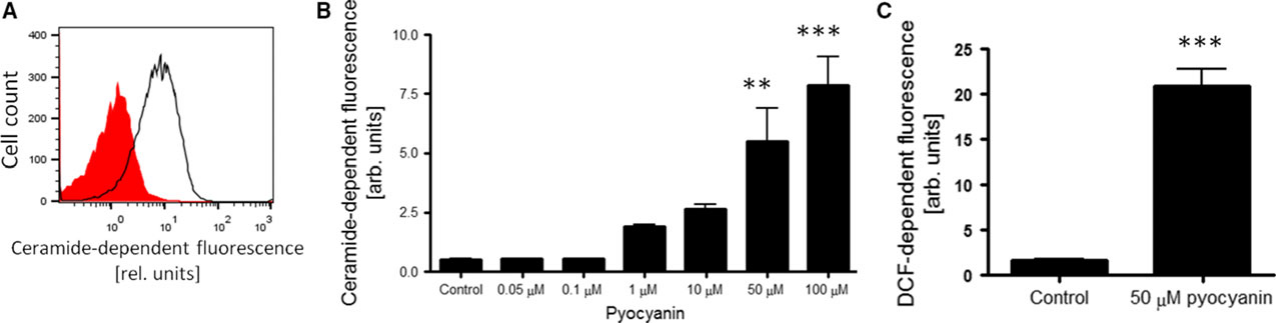
Effect of *Pseudomonas aeruginosa* pyocyanin on erythrocyte sphingomyelinase activation, caspases and generation of reactive oxygen species. Original histogram (*red shadow*: Control, *black line*: 50 μM pyocyanin; **A**) and means ± S.E.M. (**B**) of ceramide‐dependent fluorescence geomean (*n* = 4) following 48‐hr incubation with 0–100 μM pyocyanin. **,*** (*P* < 0.01, *P* < 0.001) from Control. (**C**) Means ± S.E.M. of DCF‐dependent fluorescence geomean (*n* = 4) following 48‐hr incubation with 100 μM pyocyanin. *** (*P* < 0.01) from 0 μM Control.

A further series of experiments explored the participation of additional mechanisms in pyocyanin‐induced erythrocyte death. We first examined whether pyocyanin treatment influences sphingomyelinase activation in erythrocytes, which further mediates phospholipid scrambling 26. Exposure to pyocyanin increased ceramide formation in erythrocytes, an effect reaching statistical significance at 50 μM pyocyanin (Fig. 4A and B). Ceramide formation tended to be higher at lower concentrations of pyocyanin (1–10 μM), an effect, however, not reaching statistical significance. Activation of caspases further triggers eryptosis independently of Ca^2+^ entry. To test whether caspases participate in pyocyanin‐induced eryptosis we examined the effect of the pan‐caspase inhibitor Z‐VAD‐FMK. Treatment with Z‐VAD‐FMK (10 μM) did not significantly alter pyocyanin‐induced erythrocyte PS exposure (44.7 ± 6.5%; *n* = 4) as compared to the absence of Z‐VAD‐FMK treatment (46.0 ± 6.2%; *n* = 4) suggesting that caspases do not participate in pyocyanin‐triggered eryptosis. Next, we quantified DCF‐dependent fluorescence in FACS analysis to test whether pyocyanin induces ROS production in erythrocytes. It was observed that pyocyanin treatment stimulated ROS generation in erythrocytes indicating that pyocyanin‐induced eryptosis is paralleled by redox imbalance (Fig. 4C).

**Figure 5:**
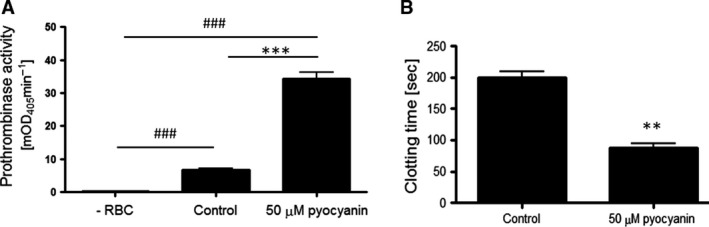
Effect of *Pseudomonas aeruginosa* pyocyanin on procoagulant activation of erythrocytes. (**A**) Means ± S.E.M. of prothrombinase activity (mOD_405_/min, *n* = 4) of erythrocytes following 48‐hr incubation in the absence (Control) or presence of 50 μM pyocyanin. As negative control, prothrombinase activity was assayed in the absence of erythrocytes (−RBC). *** (*P* < 0.001) from Control. ^###^ (*P* < 0.001) from −RBC. (**B**) Means ± S.E.M. of clotting time (*n* = 4) in plasma enriched with erythrocytes following 48‐hr incubation in the absence (Control) or presence of 50 μM pyocyanin. ** (*P* < 0.01) from Control.

Phosphatidylserine externalization is associated with procoagulant activation of erythrocytes 30. Induction of eryptosis by pyocyanin could thus confer a procoagulant phenotype on erythrocytes. To test this hypothesis we analysed the ability of pyocyanin‐treated erythrocytes to foster prothrombin activation by the prothrombinase complex. As shown in Figure 5A, pyocyanin‐treated erythrocytes significantly potentiated prothrombinase activation as compared to untreated erythrocytes. Moreover, baseline values of prothombinase activity were detected in the absence of erythrocytes (negative control) suggesting that pyocyanin treatment of erythrocytes mediates phospholipid scrambling‐dependent prothrombinase activation (Fig. 5A). To corroborate these data, pyocyanin‐treated erythrocytes were further tested for their ability to sustain coagulation in plasma using a one‐stage recalcification clotting assay. Pyocyanin‐treated erythrocytes significantly augmented clotting of plasma as compared to untreated erythrocytes (Fig. 5B). These data suggest that erythrocytes acquire a procoagulant phenotype upon exposure to pyocyanin.

**Figure 6:**
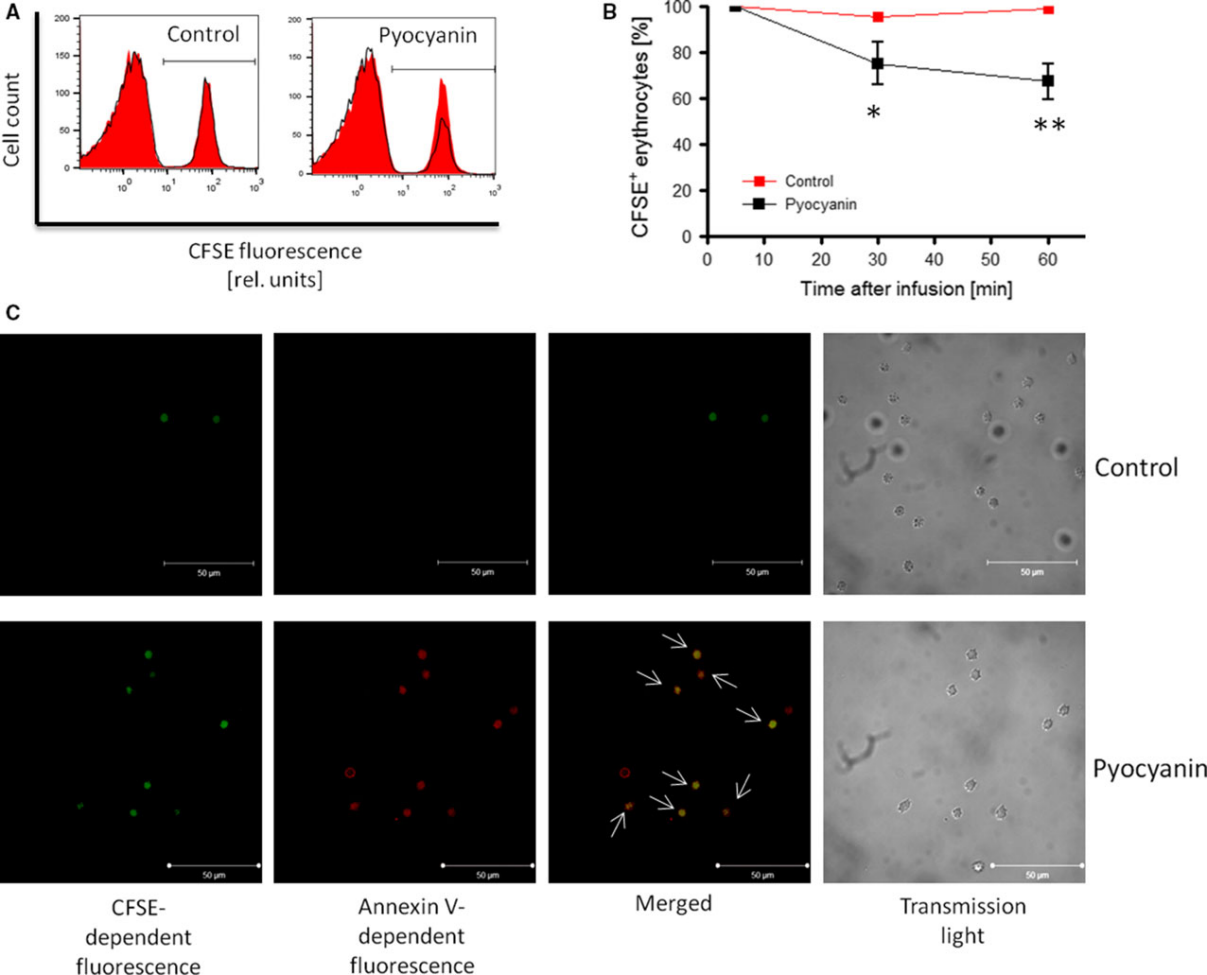
Effect of *Pseudomonas aeruginosa* pyocyanin on *in vivo* clearance of erythrocytes. Original histogram (*red shadow*: 5 min., *black line*: 60 min.; **A**) and means ± S.E.M. (**B**) of percentage of CFSE‐labelled circulating erythrocytes (*n* = 3) plotted against time after injection following 12‐hr incubation in the absence (Control) or presence of 50 μM pyocyanin. *,** (*P* < 0.05, *P* < 0.01) from Control. (**C**) Representative confocal microscopy images of CFSE‐dependent, annexin V‐APC‐dependent and merged fluorescence of erythrocytes from the spleens of mice infused with erythrocytes labelled with CFSE following 12‐hr incubation in the absence (Control) or presence of 50 μM pyocyanin. *White arrows* point to erythrocytes emitting both CFSE‐dependent and annexin V‐APC‐dependent fluorescence. For comparison, images were taken under transmission light.

Eryptosis curtails the lifespan of circulating erythrocytes by fostering their rapid clearance from the circulation 29. To test the fate of erythrocytes exposed to pyocyanin *in vivo*, murine erythrocytes treated with pyocyanin (50 μM for 12 hrs) were labelled with CFSE and infused into the circulation, and time‐dependent decay of CFSE‐positive erythrocytes was analysed. As illustrated in Figure 6A and B, the percentage of circulating CFSE‐positive erythrocytes exposed to pyocyanin was significantly diminished in 30 and 60 min. as compared to untreated erythrocytes. Cleared erythrocytes are largely retained in the spleen where they are degraded by macrophages. As depicted in the fluorescence images in Figure 6C, appreciable numbers of CFSE‐positive and annexin V positive erythrocytes were detected in the spleens of mice infused with pyocyanin‐treated erythrocytes but not in mice infused with untreated erythrocytes, suggesting that pyocyanin exposure leads to enhanced PS‐dependent clearance of erythrocytes from the circulation.

**Figure 7:**
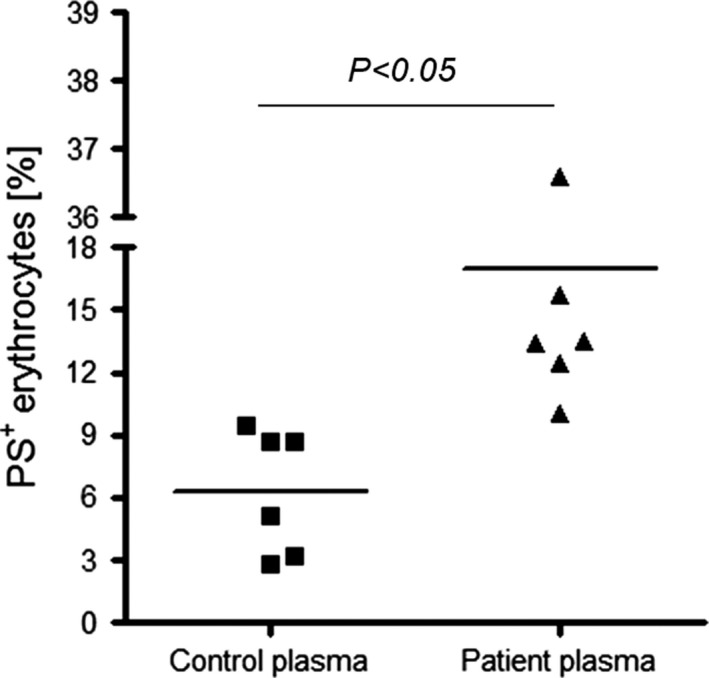
Effect of plasma from patients with *Pseudomonas aeruginosa* sepsis on phospholipid asymmetry of erythrocyte membrane. Percentage of PS exposing erythrocytes following 24‐hr incubation in plasma from healthy donors (Control; *n* = 6) or plasma from patients (*n* = 6) with *P. aeruginosa* sepsis. Each point indicates one patient plasma sample.

A further series of experiments was performed to examine the impact of *P. aeruginosa* bacteraemia on erythrocyte survival. It was found that 24‐hr incubation of erythrocytes (O^−^ blood group) in plasma from patients with *P. aeruginosa* sepsis resulted in significantly enhanced PS exposure as compared to erythrocytes incubated in plasma from healthy donors (Fig. 7). As shown in Table 2, analysis of erythrocyte parameters revealed anaemia in patients with *P. aeruginosa* sepsis as evident from significantly decreased erythrocyte count, haematocrit and haemoglobin concentration. Thus, enhanced eryptosis in *P. aeruginosa* sepsis contributes to anaemia in those patients.

**Table 2:** Erythrocyte parameters of *Pseudomonas aeruginosa* sepsis patients and healthy controls

Parameter	Control (*n* = 6)	Patients (*n* = 6)
RBC (×10^6^/μl)	4.4 ± 0.1	2.7 ± 0.1^a^
HGB (g/dl)	13.5 ± 0.5	8.8 ± 0.4^a^
HCT (%)	40.6 ± 1.7	25.0 ± 1.4^a^
MCV (fl)	91.7 ± 1.9	95.9 ± 2.4
MCH (pg)	30.6 ± 0.6	33.9 ± 1.4
MCHC (g/dl)	33.3 ± 0.2	33.6 ± 0.3

a *P* < 0.001 significant difference from healthy control.

Means ± S.E.M. of erythrocyte count (RBC), haemoglobin concentration (HGB), haematocrit (HCT), mean corpuscular volume (MCV), mean corpuscular haemoglobin (MCH) and mean corpuscular haemoglobin concentration (MCHC) determined in erythrocytes drawn from patients with *P. aeruginosa* sepsis and healthy controls.

## Discussion

This study unravels the, hitherto unknown, effect of *P. aeruginosa* pyocyanin on erythrocytes *i.e*. the stimulation of phospholipid cell membrane scrambling and cell shrinkage accompanied by enhanced cytosolic Ca^2+^ activity, ceramide formation and ROS generation. We further show that pyocyanin‐treated erythrocytes stimulate prothrombin activation and fibrin generation *via* enhanced phospholipid scrambling. In addition, we observed enhanced entrapment of pyocyanin‐treated erythrocytes in the spleen and rapid clearance from the murine circulation.

According to our observations, septic plasma from patients with *P. aeruginosa* infection triggered eryptosis. A similar effect was previously reported in erythrocytes exposed to septic plasma of different microbial aetiologies 33. Bacterial components such as peptidoglycans 35, lipopeptides 36, α‐haemolysin 37, and listeriolysin 38 were previously shown to stimulate PS exposure in erythrocytes. Sepsis‐associated eryptosis is further confounded by host factors such as histone release which was recently shown to elicit erythrocyte PS exposure 30. As a result of a multitude of virulence factors involved in *P. aeruginosa* infections, discerning the pathophysiological implication of pyocyanin alone has remained an experimental challenge. At least in theory, pyocyanin may be a contributing factor in increased eryptosis associated with *P. aeruginosa* bacteraemia. Remarkably, pyocyanin concentrations were shown to reach 130 μM in sputum from airways of cystic fibrosis patients colonized with *P. aeruginosa* 25. In rats, pyocyanin was shown to achieve a blood concentration of approximately 12 μM 39. On account of its zwitterionic properties and high diffusion potential, pyocyanin is believed to easily traverse into the systemic circulation 5. Thus, micromolar concentrations have been extensively used to study the biological effects of pyocyanin *in vitro* 10, 14, 15, 16, 18, 40, 41. The concentration of pyocyanin achieved *in vivo* during *P. aeruginosa* bacteraemia in humans, however, remains to be shown. Accordingly, substantial additional experimental effort is required to fully unravel the biological and clinical impact of inhibiting pyocyanin production *in vivo*.

Mechanistically, intracellular Ca^2+^ activity is a crucial element in eryptosis signalling 27. Our data disclose that pyocyanin potentiated enhanced cytosolic Ca^2+^ activity and stimulated calpain activation which, in turn, is a Ca^2+^‐dependent phenomenon 34. The degradation of membrane proteins by calpain fosters erythrocyte membrane blebbing, a further hallmark of eryptosis 26. In addition, ramifications of increased cytosolic Ca^2+^ activity include modification of transglutaminase 34 and cytoskeletal proteins 42. Strikingly, pyocyanin was previously shown to increase intracellular Ca^2+^ concentration in airway epithelial cells 12.

Our data show that individual batches of erythrocytes are differentially susceptible to pyocyanin which may possibly be explained by the differential age‐dependent sensitivity of erythrocytes to eryptotic stimuli 43. Remarkably, other studies have reported the presence of a considerable heterogeneity in seemingly morphologically homogenous erythrocyte populations in terms of membrane PS exposure, Ca^2+^ influx and prothrombotic activity 44. It is possible that these factors contribute to differences in the individual susceptibility of erythrocytes to pyocyanin‐triggered eryptosis. Independently of Ca^2+^ signalling, eryptosis is effectively accomplished by stimulation of erythrocyte sphingomyelinase and subsequent ceramide formation 27. We observed that pyocyanin‐induced eryptosis is paralleled by a robust increase in ceramide formation. Ceramide formation is a crucial mechanism in sepsis‐induced eryptosis 33. Intriguingly, several sepsis‐causing bacteria are known to secrete sphingomyelinases 33 that, in turn, could directly trigger ceramide formation in addition to the induction of ceramide formation by other bacterial components 35, 36. In neutrophils, pyocyanin was recently shown to induce mitochondrial ceramide formation 16. Interestingly, ceramide formation has been shown to be a decisive mechanism in the pathophysiology of *P. aeruginosa* infections and cystic fibrosis 45. Along these lines, it is, therefore, reasonable to conjecture that ceramide formation is a pivotal mechanism in pyocyanin‐induced eryptosis.

Mounting evidence suggests that pyocyanin‐induced cytotoxicity is associated with oxidative stress 18, 20. Pyocyanin treatment indeed enhanced ROS generation in erythrocytes. Oxidative stress is known to limit erythrocyte survival 46, 47, 48 due to activation of Ca^2+^‐permeable cation channels 49. Depletion of the oxidative stress scavenger glutathione renders erythrocytes vulnerable to eryptosis 50. Oxidative stress is further responsible for the activation of caspases which are, however, not required for eryptosis following Ca^2+^ entry 26. Unlike the effect of pyocyanin on nucleated cells 20, our results show that caspases do not participate in pyocyanin‐induced eryptosis. Erythrocyte survival is also influenced by a wide variety of erythrocyte‐expressed kinases such as AMPK, CK1α, PAK2 and p38 MAPK 26, 51, 52, 53. Whether those kinases participate in pyocyanin‐induced eryptosis requires further investigation.

Phosphatidylserine externalization on erythrocyte membrane serves as a platform for the assembly of the prothrombinase and tenase complexes that fosters thrombin generation and clotting 54, thus mediating the procoagulant effects of eryptotic erythrocytes 30. Our data underline that pyocyanin‐treated erythrocytes orchestrate both thrombin generation and influence clotting time. It is, therefore, tempting to speculate that pyocyanin‐induced eryptosis may participate in the thrombogenic complications of *P. aeruginosa* sepsis 32. In addition to septicaemia, eryptosis participates in the pathophysiology of a wide range of systemic conditions 26, 27, 55, 56, 57 and is associated with the toxicity of various biologically active compounds 26, 58, 59, 60, 61, 62, 63, 64, 65, 66. Furthermore, eryptosis is sensitive to erythrocyte age 67 and is an important determinant of the quality of stored erythrocytes for transfusion 68, 69.

In conclusion, this study discloses the eryptosis‐inducing effect of the virulence factor pyocyanin, thereby shedding light on a potentially important mechanism in systemic complications of *P. aeruginosa* infection.

## Conflicts of interest

The authors confirm that there are no conflicts of interest.
